# Reducing Khat use among Somalis living in Kenya: a controlled pilot study on the modified ASSIST-linked brief intervention delivered in the community

**DOI:** 10.1186/s12889-022-14681-w

**Published:** 2022-12-05

**Authors:** Marina Widmann, Bernice Apondi, Abednego Musau, Abdulkadir Hussein Warsame, Maimuna Isse, Victoria Mutiso, Clemens Veltrup, Inga Schalinski, David Ndetei, Michael Odenwald

**Affiliations:** 1 University of Konstanz, Feursteinstrasse 55, 78479 Konstanz, Germany; 2 vivo international e.V, Konstanz, Germany; 3 Voices of Community Action & Leadership, Beverly Court, Marcus Garvey Rd, Nairobi, Kenya; 4 University of Groningen, PO Box 72, 9700 Groningen, AB Netherlands; 5 Africa Mental Health Research and Training Foundation, Box 48423-00100, Matumbato Road, Nairobi, PO Kenya; 6 Tawakal Medical Clinic, 5th street, Eastleigh, Nairobi, Kenya; 7 Fachklinik Freudenholm-Ruhleben, Postfach 5, 24301, Plön, Germany; 8 Universität der Bundeswehr München, München, Germany; 9 University of Nairobi, Nairobi, Kenya

**Keywords:** Khat, Somali refugees, ASSIST, Brief intervention, Community sample

## Abstract

**Background:**

During recent decades, the consumption of the stimulant khat (*catha edulis*) has profoundly changed in countries around the Horn of Africa, and excessive use patterns have emerged—especially evident among displaced Somalis. This is related to the development of severe somatic and psychiatric disorders. There are currently no preventive or interventional studies targeting khat use. This study’s aim was to test screening and brief intervention (SBI) to reduce khat use among urban Somali refugees living in Kenya with limited access to public healthcare.

**Methods:**

In this controlled study, 330 male Somali khat users from the community were either assigned to SBI (161) or an assessment-only control condition (AC; 169); due to field conditions a rigorous experimental design could not be implemented. The World Health Organization’s (WHO) Alcohol, Smoking and Substance Involvement Screening Test (ASSIST)-linked brief intervention was adapted to khat and Somali culture. Trained local counselors administered the intervention. The amount and frequency of khat use was assessed using the time-line-follow-back method. We compared the month before the intervention (t1) to the two months after it (t2, t3). Baseline differences in khat use frequency were corrected by partial matching and mixed effect models used to evaluate intervention effects.

**Results:**

SBI was well accepted and feasible for khat users. Over the complete observation period and from t1 to t2, khat use amount and frequency decreased (*p* < .001) and the intervention group showed a greater reduction (group x time effects with *p ≤* .030). From t2 to t3, no further reduction and no group differences emerged.

**Conclusion:**

The results provide preliminary evidence that khat use amount and frequency can be reduced in community settings by SBI, requiring little resources. Thorough assessment alone might have intervention-like effects. The non-treatment-seeking community sample and the non-professional counselors are distinct from SBI studies with other substances in other countries, but support the feasibility of this approach in khat use countries and especially in Somali populations with limited access to healthcare. Future studies that employ rigorous experimental design are needed.

**Trial registration:**

ClinicalTrials.gov identifier: NCT02253589. Date of first registration 01/10/2014, retrospectively registered https://clinicaltrials.gov/ct2/show/NCT02253589. First participant 16/09/2014:

## Background

The chewing of khat leaves (*catha edulis*) is a Janus-faced practice typical of several countries around the Horn of Africa: although traditionally used for centuries among certain ethnic groups, its consumption is undergoing great changes, and it is now the main substance of abuse in several of these countries, with misuse being increasingly practiced [1, 2]. The preferred means of consumption is chewing the fresh leaves and tender stems with the effects of excitability, euphoria, talkativeness and flow of ideas. Technical progress, such as in transport infrastructure, have enabled khat production and its availability to increase significantly. Today, the khat sector is a considerable part of the national economies of Yemen, Ethiopia and Kenya, where the substance is legally traded and consumed [3, 4].

In contrast to the traditional moderate method of consumption, modern excessive patterns of use are related to various physiological and psychological health consequences [5–12]. While researchers recognized the potential of khat to induce psychological dependence early on, recent studies have confirmed this, showing the high dependence potential and high dependence rates in several populations [13–17].

Despite the great need for culturally adapted medical and psychological interventions for khat misuse and dependence, there is currently very limited knowledge available on this specific topic [2]. In one single study, psycho-education had a small effect on khat use behavior among psychotic patients in Somalia [15]. Because most countries in which khat use is common have an understaffed and over-burdened public health system, with little or no institutional care for addiction [18], most suitable interventions would be those which involve little resources and can be implemented by community health workers or in primary care settings, such as screening and brief interventions (SBI). There is strong evidence for the effectiveness of SBI in primary healthcare settings in reducing hazardous alcohol use [19]; however, the evidence for other substances is weaker [20, 21]. No specific study to date has targeted khat use interventions, and no such study has ever been conducted in community settings.

Somalia is among the world’s ‘Fragile States’ [22] and has continuously suffered from organized violence, famine and natural disasters over the last decades. By the end of 2014, more than 1.1 million Somalis were internally displaced and 970,000 were living as refugees in neighboring countries. Kenya has a strong ethnic Somali minority; additionally, more than 420,000 Somali refugees were hosted by Kenya by the end of 2014 [23]. Because of the culture of khat use, it is very likely for Somalis to chew khat to cope with adversities; it is supposed to strengthen a sense of identity and social wellbeing [24, 25]. After traumatic experiences, the amount of use was shown to be increased in order to reduce painful memories and cope with other symptoms [26]. In previous studies, extreme khat misuse patterns were found among the Somali refugee population in Kenya as well as a great need for prevention and intervention tools [16]. The World Health Organization’s (WHO) recommendation to include substance use prevention and intervention into the general healthcare [27] is problematic for urban Somali refugees in Kenya who do frequently not access healthcare services when severely ill [28]; one explanation why they avoid utilization of government-run healthcare is the missing residence permit and the associated threat of deportation [29]. Thus, alternative approaches to deliver khat-related prevention and intervention tools outside healthcare facilities are needed.

Here, we report on a pilot study that had two aims: (1) We wanted to gain initial experiences on the acceptance and applicability of interventions to reduce khat consumption in Somali culture where its use is an integral part of the contemporary life style and where at the same time misuse is increasing. We also aimed to pilot test the feasibility of the delivery of khat-related interventions in community settings because in this region healthcare settings are overburdened with already existing tasks and not frequently utilized by the target group. By that we wanted to explore new ways of reaching disadvantaged khat user groups. (2) Secondly, we wanted to generate initial data on the effectiveness of a khat version of the ASSIST-linked brief intervention (BI) that had been adapted to Somali language and culture among a non-treatment-seeking and a non-selected community sample of khat chewers. We expected that khat users from the community would reduce their khat use after receiving ASSIST-BI when compared to a control group who only participated in the repeated assessments of their substance use. We expected small reductions in the amount and frequency of khat consumption in favor of the treatment group.

## Methods

### Aim

We intended to test an adapted version of the ASSIST-linked BI on its effectiveness in a special population of khat-chewing Somali refugees and explore its acceptance and applicability in a community setting.

### Design

**Fig. 1:**
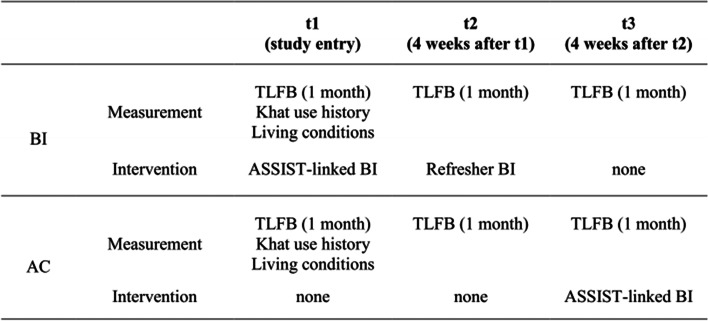
*Design of the study. At all points of assessment the measurement of khat use amount and frequency took place before delivering the intervention*

In this controlled preventive intervention study, 330 male Somali khat users from the community were assigned to Screening and ASSIST-linked brief intervention (SBI) or assessment control (AC) in an alternating order; because of the challenges met in the field (see below) this probably cannot be considered random allocation. In the course of a two-month study period, three individual appointments were scheduled: t1 at study entry, t2 four weeks after t1, and t3 four weeks after t2. Participants of the intervention group received SBI in the first appointment and a brief refresher BI at t2 appointment, asking for changes in khat use in the previous four weeks. For ethical reasons, the AC group received SBI after two months in the third appointment. See Fig. 1.

### Study setting

This study was implemented from September 2014 to December 2014 in Eastleigh, a part of Nairobi, Kenya, with approximately 300,000–500,000 inhabitants, the majority of which being ethnic Somalis, many of whom are urban refugees [30, 31]. At the time of the study, terrorist threats, security measures and gang criminality complicated the study implementation as participants and research staff had difficulty to freely move in Eastleigh.

### Sample size and eligibility criteria

Male khat chewers with an initial motivation to stop or reduce their khat use were recruited from the community. We only included male participants because khat chewing is a predominantly male habit and female users are often stigmatized in the community, which leads to significant underreporting.

Using the G*Power software [32], we calculated a theoretically needed total sample size of *n* = 328 as being sufficient to be able to detect a small within-between interaction effect (partial eta^2^ 0.01) in repeated measures ANOVA with sufficient power (ß = .95; α = .05). In the study implementation we allowed slight oversampling because we expected some drop outs.

Recruitment took place after community mobilization and information on the study, that was implemented via radio announcements, printed flyers and oral information provided by community elders and the local research team. Khat users with an interest to participate in the study were informed to directly contact the collaborating community health center for receiving more information, screening and inscription. A part of the participants were approached directly in the community by research staff members who informed on the study in mosques, community centers or other public settings; several participants could not come to the collaborating clinic for formal recruiting. Gang criminality, fear of terrorist attacks, state security measures and informal employment prevented them from leaving their location. As we used a WHO tool recommended for international use, we announced that we were looking for study participants who wanted to reduce their khat use and would accept counseling to support them. Snowball effects also occurred, as khat users included in the study motivated other fellow users to participate.

Inclusion criteria were being an adult (18–60 years) male Somali, chewing khat (within the past month), being motivated to learn about or change his khat use, while exclusion criteria were acute episodes of severe mental disorders (e.g. schizophrenia), mental disabilities and impaired communication skills. Other health conditions and other substance or medication use were no exclusion criteria. Screening for inclusion and exclusion criteria took place as second step after information of potential participants and was supported by a Somali speaking medical doctor with long-standing experience in psychiatry (AHW) who conducted the screening for exclusion criteria in the collaborating community clinic or directly supervised it by telephone calls in case the potential participant could not come to the clinic. A screening form was used as interviewer guideline with specific items related to the criteria stated above that had to be checked by the screener.

As a third step, participants were formally recruited and included by the research team by reading the study information and consent form and by signing the latter. Finally, the allocation of included participants to treatment groups was determined following a strictly alternating order by each interviewer supervised by the field research coordinator (MW). This procedure was followed with participants who were contacted in community sites and as well when recruiting interested participants in the collaborating clinic. The allocation method needed to be simple and transparent in order to gain the participant’s confidence. The research staff was supervised in the field to strictly adhere to the alternating order of allocation. In the case of cliques of khat chewers, the whole group was assigned to SBI or AC in order to minimize treatment contamination; khat users in disadvantaged settings typically spent their days together with the same group of peers and chew khat together.

Group allocation had to take place immediately after study inclusion in the field as the interviewers needed to be flexible to start the first session according to the need of the interviewee (i.e. immediate or appointment in the clinic). SBI was part of the t1 session of the intervention group. Reasons for immediate start of t1 session in the community were difficulties of the participant to relocate e.g. due to informal labor activities, fear of the participant because of insecurity in other parts of Eastleigh and difficulties with local transport.

**Fig. 2:**
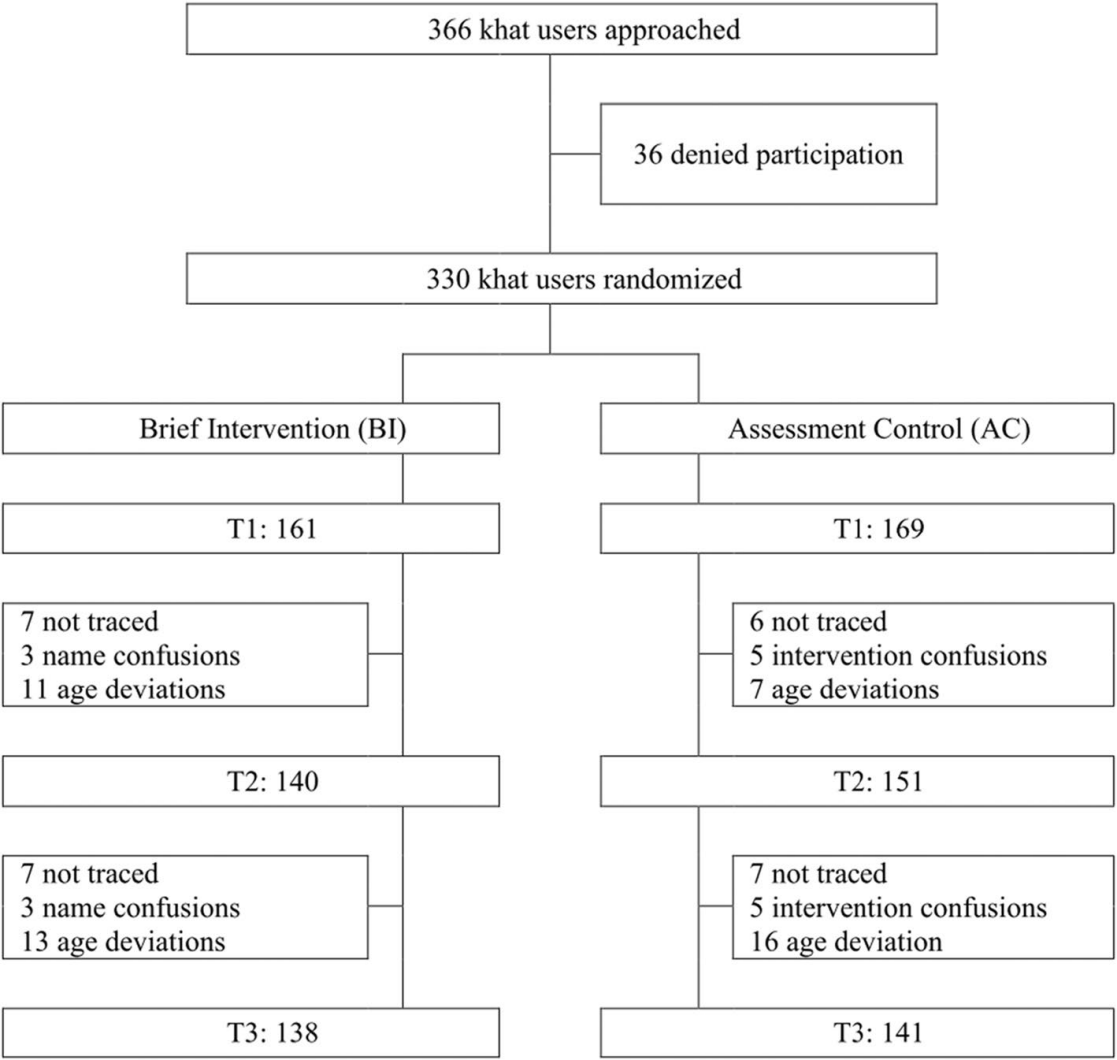
*Flow diagram*

330 participants were assigned to SBI (161) and AC (169), and an initial participation rate of 90.2% was achieved, as illustrated in Fig. 2. In order to identify individuals who took someone else’s place at a follow-up assessment, we checked socio-demographics at each assessment. The numbers of excluded participants are shown in Fig. 2. Sample characteristics are described in Table 1.

**Table 1:** *Characteristics of the study participants*

		Screening and Brief Intervention (SBI) group	Assessment control (AC) group	Total	*p*-value
*Participants (%, n)*		48.8% (161)	50.2% (169)	100% (330)	–
Socio-demographics					
Age (mean, SD)		27.7 (6.2)	28.0 (7.0)	27.8 (6.6)	.698
Marital status (% married, n)		20.5% (33)	27.2% (46)	23.9% (79)	.153
Occupation (% not working, n)		38.5% (62)	41.4% (70)	40.0% (132)	.590
Immigration status (% refugees, n)^1^		51.2% (82)	47.6% (80)	49.4% (162)	.511
Persons per household (mean, SD)		4.1 (2.7)	4.1 (2.3)	4.1 (2.5)	.950
Education, years formal school (mean, SD)		8.8 (4.4)	9.3 (3.7)	9.0 (4.1)	.267
Home type (%, n)^1^	*House*	0	1.2% (2)	0.6% (2)	.289
	*Lodge*	13.8% (22)	8.9% (15)	11.2% (37)	
	*Apartment*	69.4% (111)	72.2% (122)	70.8% (233)	
	*Part of apartment*	16.9% (27)	17.8% (30)	17.3% (57)	
Household type (%, n)^2^	*Family based*	66.2% (106)	69.3% (115)	67.8% (221)	.658
	*Rent sharing*	16.9% (27)	17.5% (29)	17.2% (56)	
	*other*	16.9% (27)	13.3% (22)	15.0% (49)	
Khat use					
Age at starting regular khat use (mean, SD)		20.6 (4.0)	21.4 (3.8)	21.0 (3.9)	.066
Other substance use last year, excluding khat and tobacco (% of participants using any other substance, n) ^3^		26.1% (42)	20.3% (32)	23.2% (74)	.217

1 missing values: SBI n = 1, AC *n* = 0.

2 missing values: SBI n = 1, AC *n* = 3.

3 missing values: SBI n = 0, AC *n* = 11.

### Instruments

#### Translation of instruments

All materials were used in Somali language after a translation and independent back-translation process by a multidisciplinary, local and international expert group. All translated materials were pilot tested within local community.

#### Substance use measures

Khat use was measured by two indicators: consumed traded units (bundles) and days with khat use in the 4 weeks prior to each assessment. We used an adapted version of the Timeline Followback [33], a calendar method with high reliability and validity [34]. In order to be able to compare the amount of use of different local khat qualities we developed, together with experienced Somali khat users and Somali physicians, a scheme of equivalent traded standard units with brief explanations, photos and local terms before the study. Based on experiences with substance users in the region the international expert group decided to retrospectively assess khat use for a period no longer than 28 days. In each time of assessment, khat use was assessed with the TLFB before the (refresher-) intervention was given and participants were not informed about the subsequent refresher BI before the TLFB was finalized.

#### Household economic situation and living conditions

In order to assess living conditions, we asked for the participant’s home type, household type, the number of people living in the household, marital status, schooling years and occupation.

### Intervention

#### ASSIST-linked brief intervention

This SBI tool consists of two parts, a screening and an intervention part, in which screening results (risk level and related health risks) are fed back to the participant. The ASSIST (*Alcohol, Smoking and Substance Involvement Screening Test)* is a brief and easy-to-apply interviewer-administered instrument developed by the WHO in multi-country studies for the identification of problematic substance use and as a starting point for a brief intervention to reduce substance use in primary care [35]. The screening tool is recommended for all cultures, is widely used and has a good to excellent reliability [36]. With eight questions on nine groups of substances, a substance involvement score and risk level for each substance (range 0–44) is assessed. In the original version, khat is not included within the predefined categories of substances and falls under “other substances”. The ASSIST-BI is a standardized brief intervention with a duration of approximately 15 minutes that builds upon substance involvement scores derived from the ASSIST interview (WHO, 2010). The intervention follows the FRAMES model, containing the six elements (feedback, responsibility, advice, menu of options, empathy, and self-efficacy) that are considered effective in reducing substance use [37], and incorporates techniques derived from motivational interviewing [38]. Additionally, a self-help booklet that transports simple information and advice by brief texts and illustrative drawings is given to the client as part of the ASSIST-linked BI. The effectiveness of the ASSIST-BI in reducing substance use has been demonstrated in a large multi-country study [35]. This SBI method has been developed to be used in primary healthcare settings all over the world and is recommended by WHO and UNODC to be applied around the globe for all substances of abuse; numerous language versions are provided on the WHO webpage.

#### Adaptation of the ASSIST and the ASSIST-linked brief intervention (ASSIST-BI)

Both tools were originally developed for intercultural use around the globe, and as recommended by the WHO, new language versions need to be adapted to specific cultural settings. A multi-disciplinary group of local and international experts reviewed and adapted the ASSIST interview and the ASSIST-BI according to the WHO’s recommendations. Because of khat being the predominant and the only culturally sanctioned substance in Somalia, they decided that the tools are applicable to khat, but that khat should be included in the list of explicitly mentioned substances (instead of falling under “others”) and that the risk feedback should be based on a risk score that is computed similar to the way used for alcohol (0–10, 11–26, 27+) rather than for illegal substances. This reflects that khat use is a legal and culturally sanctioned practice and that, in the target group, non-use leads to social exclusion.

In the present project, the ASSIST interview was only used as part of the khat-related ASSIST-linked BI because a more sensitive measure for khat use was employed; additionally, expecting only a small effect size of the intervention, mixing intervention and assessment was avoided. We assessed all questions for khat, and for all other substances, we only employed questions 1 and 2. The aforementioned expert group slightly adapted the original ASSIST-linked BI in order to fit to khat-specific and cultural requirements (i.e., to explicitly include khat in the list of substances) similarly to the way practiced in the WHO’s multi-country validation study [35]. Because khat is the main substance of abuse and is for most users the only substance of abuse, the self-help booklet was modified from a more general substance focus to a specific khat focus (i.e., the term “alcohol and substance use” was replaced with “khat use” and recommendations were modified to the Somali reality, e.g. respecting that there is limited access to leisure facilities such as bars, discotheques, cinemas or sports facilities for the target population) and translated into Somali, using the method described above.

The refresher session reviewed and reinforced the participants’ attempts to reduce khat and encouraged them to use the self-help materials.

### Training and procedures

The assessment and ASSIST-BI was implemented by a group of 15 Somali youth leaders with a college degree and 3 supervisors, all being from Eastleigh’s Somali community and fluent in Somali, Swahili and English. Prior to the study, they participated in a four-week intensive workshop and training on the theoretical background and practical aspects of BI, interviewer skills and the assessment tools, with continuous supervision during project implementation.

Assessment and interventions were conducted in Somali. Assessment and intervention were part of the same sessions. Initial sessions (t1) took place in a collaborating local health center whenever the participant would accept an appointment. In case of this being impossible (e.g. not being able to relocate because of informal labor activities, security issues or other difficulties), t1 interviews and intervention had to be performed in the field. Follow-up appointments were flexibly arranged according to the needs of participants. At any location, privacy and data protection was assured in order to be able to speak about sensitive topics. Interviewers never assessed the same respondent twice, i.e. were blinded on the previous substance use reports of the interviewed participant, but not for the group (SBI or AC) because all sessions contained specific elements so that an interviewer could conclude to which condition the participant had been allocated. Participants were not paid or compensated for their participation.

### Statistical procedures

We analyzed data using SPSS version 20. For continuous variables, we report means and standard deviations (M ± SD) and percentages for categorial variables, p was set to < 0.05.

Groups were compared with Chi^2^ tests (or Fisher’s exact tests) or with t-tests (or Mann-Whitney-U tests). For the outcome variable “number of consumed bundles”, square-root-transformed data were used due to skewed distributions. Cohen’s d was used to describe effect sizes within and between treatment groups; for between-treatment effect sizes, pre-post differences were weighted for their pooled standard deviation of the baseline measurement (d_ppc2_) [39].

#### Mixed-effect models

In order to deal with missing values, we chose mixed-effect models for the analyses of changes over time and between groups. These have the advantage of being relatively robust to missing data [40], as they allow analyses of repeated measures across time points while avoiding case-wise deletion due to missing values. The model predicts treatment response (consumed khat bundles and days with khat use) including ‘treatment’, ‘time’ and ‘treatment x time’ as fixed effects and ‘time’ as within-participants repeated factor. To correct for multiple testing, the *p*-value was set to .025 according to Bonferroni correction. The type of covariance was set as unstructured due to best model fit according to -2Log Likelihood information criteria. Random intercepts and slopes were included stepwise as subject-specific random effects to test if model fit was enhanced as recommended by Raudenbush and Bryk [41]. The model selection was based on maximum likelihood estimation but no differences in model fits for random intercepts or slopes were found, therefor the information criteria are not listed separately.

We matched the extreme 5% (*N* = 16) of the participants in order to correct for the a priori existing differences, but not to reduce the sample size too much. Using the variable “consumed khat bundles”, participants were paired (±3 bundles). This resulted in the exclusion of the 8 participants with the lowest (8 AC) and the 8 with the highest (1 AC, 7 SBI) use. In cases with more than one matching equivalent, the most similar age was decisive. The exclusion of extremes seems legitimate because various studies showed the effectiveness of brief interventions might be moderated by the amount of use [21]. After this procedure, khat use in the month before study entry, age and other socio-demographic variables did not differ between the groups.

#### Reliable change index

For individual treatment effects, we used the reliable change index (RCI) to compare treatment outcomes [42] using the test-retest reliability of the Timeline Followback for alcohol, as it is the most comparable substance regarding popularity, legal status and availability [taken from 34]. Percentages of participants with and without significant improvement (which includes change for the worse) were compared with a cut-off for significant change at RCI = 1.96 and *p* < 0.05 [42].

## Results

161 participants were assigned to the SBI and 169 to the AC group. Table 1 reports the socio-demographic and socio-economic data of both groups. They were comparable in relation to all items.

The duration of the ASSIST screening and brief intervention for the intervention group was 28.8 (±12.5) minutes at t1 and 13.1 (±8.0) minutes at t2 (refresher); in the AC group, the intervention at t3 took 38.5 (±14.9) minutes.

The experiences during study implementation showed an unexpected positive reception in the khat-using community: the study team easily recruited 330 participants out of 366 approached khat users, who motivated friends also to participate, clearly showing that counseling support to reduce khat use was greatly sought after. The dropout rates in both groups were small and comparable, supporting the high acceptance of a khat-related SBI in the target population (see Fig. 2). The feasibility of the intervention was high, as our local team had no difficulty to perform it in the Somali culture.

### Intervention effects

The quantitative indicators of khat use in the observation period (12 weeks) are reported in Table 2.

**Table 2:** *Observed amount and frequency of khat use in the 28 days prior to t1, t2 and t3*

Quantity (bundles in last 28 days; M, SD)				
time	matching	AC	SBI	*p*-value
t1	no	48.11 (43.91) *n* = 169	58.56 (48.11) *n* = 161	.006
	yes	49.40 (44.20) *n* = 160	54.37 (44.72) *n* = 154	.101
t2	no	47.75 (38.80) *n* = 155	50.75 (48.61) *n* = 143	.556
	yes	48.39 (39.03) *n* = 144	46.44 (44.36) *n* = 136	.159
t3	no	42.64 (31.75) *n* = 141	43.52 (35.94) *n* = 142	.727
	yes	42.77 (31.31) *n* = 133	42.07 (33.66) *n* = 134	.510
Frequency (use days in last 28 days; M, SD)				
t1	no	20.74 (6.28) n = 169	21.57 (6.40) n = 161	.147
	yes	21.04 (6.18) n = 160	21.44 (6.39) n = 154	.436
t2	no	18.91 (6.10) n = 155	17.19 (6.58) n = 143	.023
	yes	19.03 (5.93) n = 144	16.96 (6.57) n = 134	.008
t3	no	18.59 (7.40) n = 141	17.27 (8.22) n = 142	.100
	yes	18.81 (7.26) n = 133	17.34 (8.15) *n* = 132	.078

*p-value shows significance between AC and SBI group*

At study entry, participants in the AC and in the SBI group reported a similar number of days with khat use in the 28 days prior to the study (this did not change by partially matching the groups). In this period, participants in the AC group consumed less bundles than participants in the SBI group. With the partially matched data, this baseline difference was no longer statistically significant. Outcome of statistical testing by mixed-effect model for amount and frequency of khat use (observed means and standard deviations) are reported in Table 3.

**Table 3:** *Results of linear mixed model analyses over t1- t2- t3; t1-t2 and t2-t3*

original					matched				
Quantity (bundles)									
t		df	F	p	t		df	F	p
1-2-3	g	1, 332.63	8.66	.003	1-2-3	g	1, 317.21	3.31	.070
	t	1, 325.40	26.26	<.001		t	1, 308.48	24.89	<.001
	g x t	1, 325.40	8.23	.004		g x t	1, 308.48	4.77	.030
1–2	g	1, 331.06	9.70	.002	1–2	g	1, 317.66	4.89	.028
	t	1, 302.73	10.26	.002		t	1, 290.29	12.01	.001
	g x t	1, 302.73	6.73	.010		g x t	1, 290.29	5.40	.021
2–3	g	1, 297.13	.01	.975	2–3	g	1, 283.67	.28	.599
	t	1, 295.79	3.85	.051		t	1, 281.99	2.16	.142
	g x t	1, 295.79	.01	.935		g x t	1, 281.99	.10	.753
Frequency (days)									
t		df	F	p	t		df	F	p
1-2-3	g	1, 329.19	3.49	.063	1-2-3	g	1, 313.69	1.81	.180
	t	1, 320.51	58.93	<.001		t	1, 305.93	54.16	<.001
	g x t	1, 320.51	7.16	.008		g x t	1, 305.93	5.66	.018
1–2	g	1, 323.68	7.86	.005	1–2	g	1, 306.74	5.81	.017
	t	1, 304.83	70.94	<.001		t	1, 290.10	73.35	<.001
	g x t	1, 304.83	12.62	<.001		g x t	1, 290.10	11.76	.001
2–3	g	1, 281.30	1.49	.223	2–3	g	1, 269.38	2.32	.129
	t	1, 277.32	.03	.860		t	1, 264.35	.07	.788
	g x t	1, 277.32	.34	.560		g x t	1, 264.35	.60	.440

t = time: 1-2-3 = analysis over all 3 time points of assessment, 1–2 = analysis over time point 1 and 2, 2–3 = analysis over time point 2 and 3; df = degrees of freedom; F = F-value; p = *p*-value; g = main effect for group; t = main effect for time; g x t = interaction effect time x group

### Changes in the quantity of Khat use

**Fig. 3:**
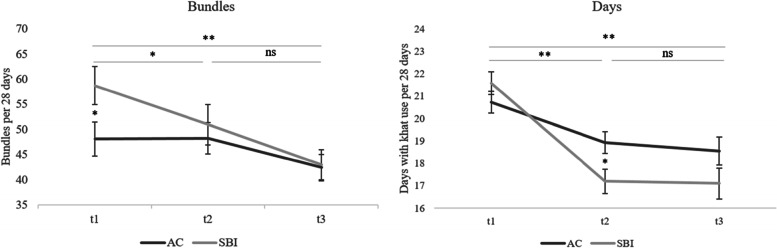
Amount and frequency of khat use with unmatched data

With the original data over all 12 weeks (t1 – t2 – t3), we found that the amount of consumed khat was reduced, with significant main effects for time and group and with a significant interaction time x group. The same was found for changes over t1 and t2, but from t2 to t3, no significant effects were found. Results with the partially matched data (t1 – t2 – t3 and t1 – t2) still showed significant main effects for time and the interaction time x group, but not for group. Observed data (not model adjusted) is reported in Fig. 3 and Table 3.

**Fig. 4:**
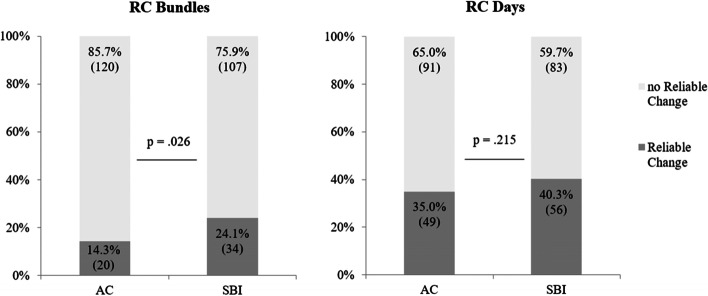
Reliable Change Index for bundles and days using unmatched data. Percentages and n of participants with and without significant reduction at t3 compared to t1

Using the unmatched data, a significant change on an individual level was assessed using the reliable change index. In the SBI group, more participants significantly reduced the amount of consumed khat bundles (t1 to t3) compared to AC; χ^2^ (1, *N* = 281) = 4.37, *p* = .026 (see Fig. 4).

The pre-post effect size (t1 – t3) for the SBI group was d = .36, for the AC group d = .14 and the between-group effect size was d_ppc2_ = .21. After the partial matching, it was d = .19 for the AC, d = .34 for the SBI and d_ppc2_ = .13 between the groups.

### Changes in the frequency of Khat use

Using the unmatched data to test the changes in the frequency of khat use, the main effect for time and the interaction time x group were significant, but not the main effect for group (t1 – t2 – t3 and t1 – t2). From t2 to t3, none of the effects were significant (see Table 3 and Fig. 3).

Using the partially matched data, the same effects remained significant (see Table 3).

Using the unmatched data with the reliable change index, no significant change could be found for the number of days with khat use in the last month, χ^2^ (1, *N* = 279) = .831, *p* = .215 (Fig. 4).

With the unmatched data, the effect size (t1 – t3) for the SBI group was d = .62, for the AC group d = .32 and the between-group effect size was d_ppc2_ = .34. After the partial matching, it was d = .34 for the AC, d = .58 for the SBI and d_ppc2_ = .29 between the groups.

## Discussion

The present study demonstrated the feasibility and provided preliminary data on the effectiveness of a khat-specific screening and brief intervention in male Somali with limited access to public healthcare from a community in Eastleigh/Nairobi.

Male Somali khat chewers from the community in Eastleigh/Nairobi participated in a khat-specific screening and brief intervention study (one session of modified ASSIST-linked brief intervention plus refresher vs. assessment only) with a 2-month follow-up. Due to pre-existing group differences, we partially matched the groups for statistical testing. Screening and brief intervention for khat use was well accepted and could be easily applied in Somali culture, as shown by this study’s ability to recruit and retain participants as well as through the application of the FRAMES model during training and study without any difficulties. It was evident that today, the wish to reduce one’s own khat use is clearly present in the Somali community in Nairobi. This matches with several recent studies from Ethiopia that found approximately 70% of khat chewers had the intention to stop khat chewing [43]. These results support the idea that focusing on khat users in community settings to deliver SBI for khat is a feasible approach to reach disadvantaged groups who do not utilize government-run healthcare. The main empirical findings of this study were that khat use amount and frequency were reduced in the intervention group vs. assessment-only control in the first month, but no differences were found in the second month; due to methodological problems, this result needs to be considered preliminary evidence for the effectiveness of SBI for khat. This result shows, however, the positive potential of very brief psychotherapeutic interventions, even if conducted by trained laypersons, in non-medical settings and with non-treatment-seeking participants from the community in the Somali khat culture, in which khat is integral part of daily life. These results encourage to conduct further research on the SBI approach because there isn’t any controlled empirical research on khat use interventions and observational studies show the difficulties to maintain abstinence without professional support [44].

In this study, we dealt with a form of substance use that is strongly related to a defined geographical region, specific ethnic groups and cultures, and which has a longstanding tradition and use culture as well as numerous social functions [45]. It is likely that members of the Somali community in Nairobi use khat to strengthen group identity and to cope with various stressors and psychopathology [5, 24–26, 46, 47]. It is almost impossible for male Somalis to participate in social life without chewing khat: their physical and social environments are full of khat-related triggers and role models aggravated by the lack of alternative leisure activities. It is equally likely that members of the Somali community use khat to cope with past and current adversities, such as those related to every-days experiences of discrimination and the illegal immigration status. This background is important when discussing intervention effects. Nevertheless, khat misuse patterns develop swiftly in this group, causing a lot of harm, and prevention and intervention concepts are urgently needed. Our pilot study shows that there is potential to develop feasible and effective concepts for prevention and intervention that require little resources and can be implemented sustainably in the community without adding another task to the already over-burdened healthcare system.

In the reported study, the preliminary effects of SBI on khat use were small; that is, at the end of the observation period the intervention group had reduced the amount by more than 10 khat bundles more (absolute change calculated from unmatched observed data; baseline range 8 to 336 bundles in 28 days; AC mean 48.11 (±43.91), SBI mean 58.56 (±48.11)) and the frequency by more than two days more (absolute change calculated from unmatched observed data; baseline range 4 to 28 days; AC mean 20.74 (±6.28), SBI mean 21.57 (±6.40)) than the control group. These differences are indeed small, and only reached significance for use frequency. When compared to previous studies, especially in non-western settings, larger effect sizes were reported [35]. These small effects can be explained by some typical features of khat use and by some characteristics of our study: compared to conventional intervention studies, we set the inclusion criteria very low and aimed for a representative community sample instead of a treatment-seeking sample. As a consequence, we included users with a wide range of consumption patterns, from addictive and excessive use to moderate and occasional use. Most other SBI studies pre-selected participants and included only those with moderate use, recruited from primary care facilities and with probably higher levels of motivation to change. Because of these factors, we had to deal with large variances in our two central outcome measures (amount and frequency), which made it difficult to identify small changes by methods of statistical testing. The variance of our measures of amount of khat use is large, especially because of the local definition of a standard unit: a relatively small quantity that easily varies at the individual level between one and ten per day. Additionally, we employed measures to retain participants in our study—even those with minimal motivation to change—such as methods to minimize the effort for participants to keep the follow-up appointments by, for example, setting very flexible times and locations, and we had two staff members from the community to motivate participants. This led to very low dropout rates, and even participants with low interest and motivation completed the study, which of course weakened the effects. Furthermore, the unselected community sample presented another problem: comorbidity with other mental disorders. In another publication on these data [48], we showed a very high prevalence of depressive, posttraumatic and psychotic symptoms in this sample, and that co-morbidity decreased the effects of SBI. Here, we were not focusing on comorbidity and did not control for it. Still, we found preliminary evidence for a reduction in khat use by SBI in the whole sample.

Additionally, we have to take into account a variety of factors that might have diminished the difference between the intervention and control conditions: First, the control group also showed intervention-like effects, as revealed by the course of change (Fig. 3). Especially in the first month, earlier reduction occurred in the SBI group, and the control group partially caught up in the second month. The overall reduction in khat use might have various reasons: The assessment of khat use and related problems (e.g., comorbid psychopathology) could have worked as intervention-like elements, comparable to the balancing exercise of MI. All these assessment components can create *discrepancy* and *change talk*. In other studies, control groups have also shown a reduction in their substance use [49]. Another reason for the small difference between intervention and control conditions might have been the counselors: trained young college graduates are not trained health workers, for whom the ASSIST-linked BI was originally developed. They had probably not performed the intervention as effective and might have not managed to deliver the *difference* between the two arms—treatment fidelity—as experienced health staff.

But in sum, preliminary effect size measures showed the expected small treatment effects (Cohen’s d = .29 and d = .13) favoring SBI, and we believe that the aforementioned factors all increased the external validity of the study and show the potential of SBI to reduce khat use in the community.

This study also needs to be discussed in the context of the growing evidence that SBI works for alcohol, but that its effectiveness for illegal substances is seriously questioned [21]. Richard Saitz [21] discussed potential explaining factors, such as non-legal status or comorbid use of other substances among drug users. In the Somali community in Nairobi, khat use has some features that are typical for alcohol use in western countries: khat is a fully legal substance; it is normal to use khat, and chewing is even considered part of the mainstream Somali culture; and in contrast to other substances, moderate Islamic currents sanction khat use. Though we found relatively low comorbid substance use, in contrast to representative samples [50], khat use in Eastleigh has also some similarities to illegal drug use in Western countries, such as the amphetamine-like effects and the typical use culture. Thus, khat chewing cannot be easily classified along the distinction between alcohol and illegal substances in western countries.

### Limitations

The study setting was extremely challenging: gang criminality, threat of terrorism and the current political situation led to high tension in Eastleigh by the time of assessment. Therefore, the implementation of a high-standard methodology was not always possible, and continuous supervision could not be maintained. Nevertheless, we believe that it is crucial to conduct such studies despite the difficult circumstances in order to develop intervention tools for extremely neglected populations.

The validity and reliability of the ASSIST and ASSIST-linked Brief Intervention for khat need further study in the future; this pilot study showed preliminary evidence and encourages the allocation of scientific attention and resources for it but further stringent research is needed before it can be considered a valid and effective measure.

We used convenience and snowball sampling as it is often the case in treatment studies. As an unintended consequence, participants who were assigned to the intervention group probably had a higher motivation to recruit friends (in order to help them). We believe that it was especially more likely for heavy chewers who received the intervention to recruit fellow users than participants from the control group. However, in total, only a small proportion of participants were recruited in that way (total snowballed proportion approximately 15–20%, including approximately 10 heavy chewers), so that we believe this could be effectively dealt with.

The allocation strategy used in our study partially failed to produce two fully comparable subgroups, as the SBI group showed greater baseline khat use than the control group. This is the consequence of a study carried out under extreme conditions in difficult surroundings, e.g. that participants and interviewers could not freely move. But after matching the extreme ends, groups were comparable. These baseline differences were probably the consequences of our allocation method, assigning every other person to the intervention group, that also included assignment of whole cliques to one condition in order to minimize contamination.

In sum, the extreme setting forced us to implement a locally tailored allocation procedure that was not a sophisticated random allocation, and at the same time, we tried to minimize selection and performance bias by some measures.

Although the motivation to stop or reduce khat use was a pre-requisite for participation, this variable was not measured. Because of the complexity of this construct and the difficulty to express it in Somali culture, the development of an instrument to measure it would require a project of its own. But this omission causes uncertainty about a-priori group differences. Future studies need to develop methods to measure this variable and clarify its influence on the intervention effects.

The study can be criticized because of non-objective measures at different steps. Inclusion and exclusion criteria were assessed without biological measures and structured clinical interviews. We had no objective substance use outcome measures such as urine screenings, partly because of missing screening tools for khat and other substances that are applicable under field conditions. However, because of the setting and culture, comorbid substance use was probably not as large as in typical studies among substance users. Therefore, we did not control for other substance use in this sample. Self-report as substance-use measure could potentially lead to underestimation. As there was no advantage for participants in underreporting, we think, in this study setting the underestimation of khat use is not substantial and therefore only a minor limitation for the interpretation of the results.

Furthermore, the missing blindness of interviewers for group allocations could have caused assessment effects in the sense that interviewer behavior could have induced more socially desirable answers. However, in order to minimize this potential effect, we strictly organized the study in such a way that no interviewer saw a participant more than once, limiting possible interviewer biases.

In sum, this controlled study was a pilot research that encourages subsequent trials with better designs to definitively determine the effectiveness of SBI with khat users.

## Conclusions

Based on our experiences from this and other projects with Somalis, we conclude that psychotherapeutic measures can be culturally accepted and applicable among this extremely disadvantaged population and in the difficult setting of the countries around the Horn of Africa and delivered in community settings instead of the already overburdened primary healthcare centers. These results provide initial support for the applicability and effectiveness of an economical prevention and intervention that requires little investment in time and training. It might have a great potential to change khat use on a broader scale. It is justifiable to allocate further resources to the development, evaluation and implementation of khat treatment and prevention programs. There is great demand for such programs in the traditional khat-using countries, in which these are completely lacking.

## Data Availability

Data cannot be shared publicly because they contain sensitive medical information. The data underlying the results presented in the study are available upon reasonable request from the corresponding author.

## Abbreviations

AC: Assessment control group
ANOVA: Analysis of variance
ASSIST: Alcohol, Smoking and Substance Involvement Screening Test
BI: Brief intervention
FRAMES: Feedback, responsibility, advice, menu of options, empathy, self-efficacy
GLMM: General linear mixed models
M: Mean
RCI: Reliable change index
SBI: Screening and brief intervention
SD: Standard deviation
TLFB: Timeline Followback
UNODC: United Nations Office on Drugs and Crime
WHO: World Health Organization.
